# In vitro fertilisation with recombinant follicle stimulating hormone requires less IU usage compared with highly purified human menopausal gonadotrophin: results from a European retrospective observational chart review

**DOI:** 10.1186/1477-7827-8-137

**Published:** 2010-11-08

**Authors:** Geoffrey H Trew, Adam P Brown, Samantha Gillard, Stuart Blackmore, Christine Clewlow, Paul O'Donohoe, Radoslaw Wasiak

**Affiliations:** 1 Consultant in Reproductive Medicine and Surgery, Hammersmith Hospital, London UK; 2 Abacus International, Manchester, UK; 3 Abacus International, Bicester, Oxfordshire, UK; 4 Formerly from Merck Serono, Feltham, Middlesex, UK; 5 Merck Serono, Bedfont Cross, Stanwell Road, Feltham, Middlesex, TW14 8NX, UK; 6 United BioSource Corporation, London, UK

## Abstract

**Background:**

Previous studies have reported conflicting results for the comparative doses of recombinant follicle stimulating hormone (rFSH) and highly purified human menopausal gonadotrophin (hMG-HP) required per cycle of *in vitro *fertilisation (IVF); the aim of this study was to determine the average total usage of rFSH versus hMG-HP in a 'real-world' setting using routine clinical practice.

**Methods:**

This retrospective chart review of databases from four European countries investigated gonadotrophin usage, oocyte and embryo yield, and pregnancy outcomes in IVF cycles (± intra-cytoplasmic sperm injection) using rFSH or hMG-HP alone. Included patients met the National Institute for Health and Clinical Excellence (NICE) guideline criteria for IVF and received either rFSH or hMG-HP. Statistical tests were conducted at 5% significance using Chi-square or t-tests.

**Results:**

Of 30,630 IVF cycles included in this review, 74% used rFSH and 26% used hMG-HP. A significantly lower drug usage per cycle for rFSH than hMG-HP (2072.53 +/- 76.73 IU vs. 2540.14 +/- 883.08 IU, 22.6% higher for hMG-HP; p < 0.01) was demonstrated. The median starting dose was also significantly lower for rFSH than for hMG-HP (150 IU vs. 225 IU, 50% higher for hMG-HP, p < 0.01). The average oocyte yield per IVF cycle in patients treated with rFSH was significantly greater than with hMG-HP (10.80 +/- 6.02 vs. 9.77 +/- 5.53; p < 0.01), as was the average mature oocyte yield (8.58 +/- 5.27 vs. 7.72 +/- 4.59; p < 0.01). No significant differences were observed in pregnancy outcomes including spontaneous abortion between the two treatments. There was a significantly higher rate of OHSS (all grades) with rFSH (18.92% vs. 14.09%; p < 0.0001). The hospitalisation rate due to OHSS was low but significantly higher in the rFSH group (1.07% of cycles started vs. 0.67% of cycles started with rFSH and hMG-HP, respectively; p = 0.002).

**Conclusions:**

Based on these results, IVF treatment cycles with rFSH yield statistically more oocytes (and more mature oocytes), using significantly less IU per cycle, versus hMG-HP. The incidence of all OHSS and hospitalisations due to OHSS was significantly higher in the rFSH cycles compared to the hMG-HP cycles. However, the absolute incidence of hospitalisations due to OHSS was similar to that reported previously. These results suggest that the perceived required dosage with rFSH is currently over-estimated, and the higher unit cost of rFSH may be offset by a lower required dosage compared with hMG-HP.

## Background

The prevalence of infertility has increased over the past 20-30 years, in part due to the rise in obesity levels [1] and a decline in male fecundity and poor semen quality [2] but also due to couples choosing to have children later in life [3]. The reported prevalence of infertility in Europe varies significantly between studies, and there is a paucity of reliable data. A recent review of international infertility rates reported the current median prevalence of infertility to be 9% over 12 months among women aged 20-44 in married and consensual unions [4]. The proportion of infertile couples seeking infertility treatment in more developed countries ranged from 42-76% [4]. A recent report from the Human Fertilisation and Embryology Authority (HFEA) states that in the year 2007, 36,861 women in the United Kingdom (UK) received 46,829 cycles of IVF treatment; a 5.8% increase from 2006 [5].

In conventional *in vitro *fertilisation (IVF) treatment, gonadotrophins are administered in order to stimulate an ovarian cycle. Follicle stimulating hormone (FSH) is universally recognised as the key driver of ovarian follicle growth and maturation, and is most often administered in one of two forms: recombinant FSH (rFSH; Gonal-F^® ^(Merck Serono) or Puregon^® ^(Merck Sharp and Dohme)) or highly purified human menopausal gonadotrophin (hMG-HP; Menopur^® ^(Ferring) or Merional^® ^(Pharmasure)) which contains both FSH and luteinising hormone (LH) activity in a ratio of 1:1.

The comparative clinical efficacy of rFSH and hMG and/or hMG-HP has been investigated previously. A Cochrane review published in 2003 [6] concluded that there was insufficient evidence to establish a difference between hMG and rFSH on ongoing pregnancy rate or live birth rate for ovulation stimulation in IVF or ICSI cycles. More recent meta-analyses have included the results from more recent studies including the MERIT study group [7]; a large randomised controlled trial (RCT) that compared hMG-HP with rFSH in patients undergoing IVF/ICSI. Two of these meta-analyses, which evaluated the comparative efficacy of rFSH and hMG (both of which included hMG-HP and hMG) for ovarian stimulation in IVF or ICSI cycles, reported a higher rate of clinical pregnancy and live birth rate for hMG than rFSH [8,9]. An additional meta-analysis (which included hMG-HP only) [10], concluded that there was no statistical difference between rFSH and hMG-HP in ongoing and live birth rates in a combined group of IVF and ICSI; however, subset analysis showed a statistically significant higher ongoing pregnancy/live birth rate in favour of hMG-HP in IVF cycles. This study also reported the total dose (International Units, IU) of gonadotrophins used and the average number of oocytes retrieved; there was significant statistical heterogeneity between trials, but sensitivity analyses suggested that there was no significant difference in these measurements between treatments.

There is a perception that the cost per IVF cycle with rFSH is more expensive compared with the Menopur^® ^brand of hMG-HP, as the 75 IU equivalent of Gonal-F^® ^rFSH has a higher unit cost than the corresponding price for Menopur^®^. It is unclear whether the same dose (total IU dose) of rFSH and hMG-HP is required per IVF cycle; however, several studies suggest that this may not be the case. While meta-analyses by Coomarasamy et al [9] (which included hMG as well as hMG-HP) and a recent study by Al Inany et al (2009) (which included hMG-HP only) [10] showed no significant difference in IU requirement between the products, an earlier meta-analysis by Al-Inany et al (2008) [8] (which included both hMG and hMG-HP), did show a reduced IU requirement for rFSH compared with hMG/hMG-HP. In addition to this, the recent large MERIT randomised controlled trial (RCT) [7] has reported a reduced IU requirement per cycle for rFSH versus hMG-HP.

The total dose of gonadotrophin per IVF cycle and its associated cost may be a factor in determining which gonadotrophin clinicians and payers choose to use in their clinic. Therefore, it is important to understand the comparative dosing when establishing the relative costs of two different treatments. Two studies have reported the comparative cost per IVF cycle using fresh embryo transfer with rFSH and hMG-HP [11,12] and both conclude that treatment per IVF cycle with hMG-HP is ultimately less costly than treatment with rFSH (based on fresh embryo transfer). In these studies, both rFSH and hMG-HP were administered at the same starting dose (IU) and titrated according to treatment outcomes. If it is true that a lower than anticipated mean IU dosage of rFSH is required to achieve comparable outcomes to hMG-HP, it can be hypothesized that the net cost of treatment with rFSH may be comparable or lower.

The objective of this study was to determine the average total IU usage in IVF cycles using rFSH alone versus those utilising hMG-HP alone in combination with long protocol GnRH agonist for pituitary down-regulation. This large retrospective chart review from four European countries provides an insight into the use of gonadotrophins in the 'real-world' clinic setting, as opposed to the data gathered from RCTs which are limited by protocol-driven treatment regimens and often may not reflect 'real-world' clinical practice. In the absence of European consensus guidelines for IVF treatment, the data included in this review were selected in a strict and robust method according to the patients' eligibility for IVF treatment as defined by the current NICE guidelines [13]. NICE are a globally recognised organisation for guideline production and a robust source of information. Their advice is often consulted and adopted by other countries in the absence of local guidelines. This study did not focus on the costs of comparative treatments, or the economic implications of the results; therefore, no cost data are reported here.

## Methods

### Study design

This study was an observational retrospective chart review of anonymised patient records from female patients who have received rFSH or hMG-HP for IVF (±ICSI) in either cycles 1, 2 or 3, across centres in Spain, Germany, Denmark and Switzerland. Anonymised data relating to gonadotrophin usage (type and amount administered), oocytes retrieved, embryos transferred, embryos cryopreserved, the occurrence of ovarian hyperstimulation syndrome (OHSS) and pregnancy outcomes were collected retrospectively from clinic records, for all eligible patients undergoing treatment. The severity of OHSS is classified according to three grades of severity: mild, moderate and severe, as defined by the World Health Organisation (WHO) [14]. OHSS is classified according to the degree of abdominal distension, ovarian enlargement, and respiratory, haemodynamic, and metabolic complications. Only cycles from patients who would be eligible for treatment under the current National Institute for Health and Clinical Excellence (NICE) guidelines [13] were selected.

Eligible cycles were extracted from raw data sets provided by four European countries. To be eligible for inclusion in the review, the cycles must have met the following criteria:

Cycles that were:

(i) 1st, 2nd or 3rd IVF (±ICSI) cycles in women aged 23-39 years at the start of treatment with a body mass index (BMI) of ≥19 and ≤29.

(ii) In women who have received a single form of FSH only in a given cycle.

(iii) In women who have received rFSH only (Gonal-F^® ^or Puregon^®^) or hMG-HP only (Menopur^® ^or Merional^®^) at a starting dose of ≤300 IU FSH/day.

(iv) Cycles with long protocol, subcutaneous, GnRH agonist down-regulation.

(v) In indications labelled in the Summary of Product Characteristics (SPC) for the product.

(vi) Administered in 2004 or later with gonadotrophin treatment for ≤25 days per cycle.

Cycles were excluded if:

(i) The patient has had 3 or more previously stimulated, unsuccessful cycles of IVF (± ICSI).

(ii) The patient was ≥40 years old at the start of treatment.

(iii) Down-regulation protocol was not long protocol, subcutaneous GnRH agonist.

(iv) More than one FSH-containing product was used in a single cycle.

(v) Follicle stimulation was achieved with a combination of FSH and another adjunctive ovary stimulating product.

(vi) The starting dose of gonadotrophin was >300 IU per day in order to exclude poor responders.

(vii) The indication was not licensed in the SPC for the product.

(vii) Treatment was performed prior to 2004 (2004 was used as a cut off date, as cycles prior to 2004 may include non-highly purified HMG).

(xi) The number of treatment days was >25.

### Study endpoints

The primary endpoint of the study was the average IU usage of rFSH versus hMG-HP per IVF cycle, utilizing long protocol GnRH agonist for pituitary down-regulation. The mean and median starting dose of each gonadotrophin was also determined. Secondary clinical endpoints included (i) oocyte yield (ii) mature oocyte yield (iii) the average number of oocytes/embryos frozen per cycle, following IVF treatment with rFSH versus hMG-HP. Tertiary endpoints included the number of IVF cycles resulting in (i) clinical pregnancy (ii) ongoing pregnancy (iii) spontaneous abortion.

The main safety endpoints of the study include (i) the rate of ovarian hyperstimulation syndrome (OHSS), (ii) rate of OHSS resulting in hospitalisation (iii) IVF cycle withdrawals.

In addition to the overall analysis, cycles were also stratified according to the patient's age at the time of IVF treatment (≤35 years and ≥36 years).

### Analysis of the raw data

**Figure 1:**
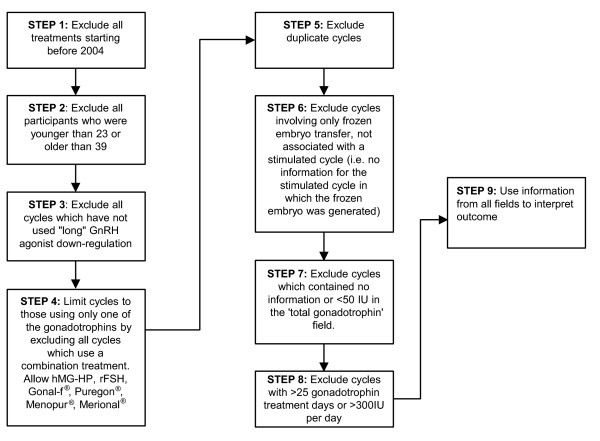
**Algorithm flow process**.

An algorithm was developed which extracted for further analysis those treatment cycles meeting the inclusion/exclusion criteria. The algorithm consisted of nine steps (Figure 1) and was validated using two different methods. Firstly, the algorithm extracted data that compared to the results of hand-triaged Spanish data. Individual cycles in the raw Spanish data set were examined individually and only those cycles which fulfilled the entry criteria were extracted for further analysis. This step led to refinements of the algorithm. In addition, each step of the algorithm was run separately to examine which data were included and excluded and the findings were cross-checked to ensure that the algorithm includes or excludes relevant cycles.

### Statistical analysis

Distributions of categorical variables were described in terms of the number and proportion of cycles in each level of the variable. For continuous data variables, the results were presented as the mean, standard deviation, median and range of values.

With regard to the association between IU per cycle and outcome variables the approach depended on whether the outcome was categorical (has at least one procedure during the study period), or continuous (has a number of procedures during the study period). For categorical variables, the outcome was cross-tabulated by sub-groups of interest, to describe the proportion in each level having the outcome and tested using a Chi-square test. For continuous variables, the distribution of the values of the outcome was described for sub-groups of interest. If the outcome variable had a normal distribution, a t-test or ANOVA was used to test the association. If the distribution of the outcome variable was skewed, a log-transformation was applied to correct the distribution. If this failed, non-parametric tests like the Mann-Whitney or Wilcoxon (Kruskal-Wallis) tests were applied.

All statistical tests were conducted at 5% level of significance. Unless otherwise stated statistical tests were two-sided. Analyses were carried out using SAS software version 9.1 (SAS Institute Inc, North Carolina, USA).

## Results

### Baseline

Following triage of the raw data using the algorithm, a total of 30,630 IVF cycles (35.6% of the total cycles) were eligible for inclusion in the review, 74% of which involved treatment with rFSH and 26% with hMG-HP. Demographics and baseline characteristics are shown in Table 1. Although there was a statistically significant difference in patient age between treatment groups (p < 0.01), the difference in age was 0.15 years (and the mean patient age was 32.93 years) and therefore it was not deemed to be clinically significant. Of the cycles included in the analysis, 20,564 cycles were conducted in patients ≤35 years and 10,066 were conducted in patients ≥36 years (67% and 33% of cycles, respectively). All patients included in the study were <40 years of age.

**Table 1:** Baseline characteristics

Characteristic	rFSH	hMG-HP	All Patients
Number of cycles			
All patients N (%)	22665 (74%)	7965 (26%)	30630
≤35 years N (%)	15422 (75%)	5142 (25%)	20564
≥36 years N (%)	7243 (72%)	2823 (28%)	10066
Age Mean (SD)	32.89 (3.57)	33.04 (3.71)^1,2^	32.93 (3.61)

1 p < 0.01 in Wilcoxon two sample test

2 p < 0.01 in two sample t-test

The vast majority of the chart data (>90%) was from centres in Germany (Table 2); however, data from all included countries were pooled for the purpose of the analysis.

**Table 2:** IVF cycles according to country

Characteristic		rFSH	%	hMG-HP	%	All Patients
Denmark	N	1027	97.5	26	2.5	1053 (3.4% of total)
Germany	N	20144	71.78	7919	28.22	28063 (91.6% of total)
Spain	N	485	99.8	1	0.2	486 (1.6% of total)
Switzerland	N	1009	98.15	19	1.85	1028 (3.4% of total)
**Total number of cycles**	**N**	**22665**	**74.0**	**7965**	**26.0**	**30630**

### Mean usage and duration of treatment with rFSH versus hMG-HP

The mean total number of IU of rFSH used per cycle of IVF (± ICSI) treatment was significantly lower than that for hMG-HP (2072.53 ± 768.73 IU vs. 2540.14 ± 883.08 IU, p < 0.01) (Table 3). The mean usage of hMG-HP was 22.6% greater than that for rFSH. In addition to this, the mean length of gonadotrophin treatment per IVF cycle was significantly shorter with rFSH than hMG-HP (11.41 ± 2.24 days vs. 11.65 ± 2.42 days, p < 0.01), but this difference is not clinically relevant. The mean starting dose of treatment was significantly lower for rFSH than for hMG-HP for all patients (176.38 ± 50.01 IU vs. 206.16 ± 55.64 IU, p < 0.01), as was the more clinically relevant median starting dose (150 IU vs. 225 IU, p < 0.01) (Table 3). Significant differences in the primary outcomes were also observed when the data were stratified according to patient age at time of treatment (Table 3).

**Table 3:** Primary outcomes

Characteristic	rFSH	hMG-HP	p-value*	All Patients
Mean (SD) total dose of gonadotrophin per IVF cycle (IU) (All patients)	2072.53 (768.73)	2540.14 (883.08)	<0.01	2194.13 (825.9)
(≤35 years)	1974.90 (726.77)	2418.56 (834.46)	<0.01	2085.84 (779.18)
(≥36 years)	2280.41 (813.09)	2761.60 (925.33)	<0.01	2415.36 (873.21)
Mean (SD) total days of gonadotrophin treatment per IVF cycle (All patients)	11.41 (2.24)	11.65 (2.42)	<0.01	11.47 (2.29)
(≤35 years)	11.41 (2.28)	11.65 (2.48)	<0.01	11.47 (2.33)
(≥36 years)	11.41 (2.16)	11.65 (2.31)	<0.01	11.48 (2.21)
Mean (SD) starting dose of gonadotrophin (IU) (All patients)	176.38 (50.01)	206.16 (55.64)	<0.01	184.12 (53.16)
(≤35 years)	167.97 (46.65)	195.94 (53.48)	<0.01	174.97 (49.94)
(≥36 years)	194.26 (52.18)	224.76 (54.67)	<0.01	202.82 (54.63)
Median starting dose of gonadotrophin (IU) (All patients)	150	225	<0.01	150
(≤35 years)	150	225	<0.01	150
(≥36 years)	187.5	225	<0.01	225

* Statistical significance for all outcomes assessed via Wilcoxon two sample test and two sample T-test

### Mean oocyte yield after treatment with rFSH versus hMG-HP

A statistically significant higher proportion of hMG-HP cycles resulted in oocyte retrieval compared with rFSH cycles (99.13% for rFSH vs. 99.91% for hMG-HP; p < 0.0001) (Table 4); however, it should be noted that the German dataset did not include cycles where no oocytes were retrieved. The mean oocyte yield per IVF cycle in patients treated with rFSH was significantly greater than in patients treated with hMG-HP (10.80 ± 6.02 vs. 9.77 ± 5.53; p < 0.01), with an increase of 10.5% seen for rFSH (equivalent to one extra oocyte per cycle on average). The mean mature oocyte yield was also significantly higher for rFSH than hMG-HP (8.58 ± 5.27 vs. 7.72 ± 4.59; p < 0.01) with an 11.1% increase in yield with rFSH, but this difference is not clinically significant. The mean oocyte yield and mean mature oocyte yield were significantly higher for patients treated with rFSH in both the ≤35 years and ≥36 years age subgroups (Table 4).

**Table 4:** Secondary outcomes

Characteristic	rFSH	hMG-HP	p-value	All Patients
Mean (SD)oocyte yield (number of oocytes retrieved) (All patients)	10.80 (6.02)	9.77 (5.53)	<0.01	10.53 (5.91)
(≤35 years)	11.21 (6.11)	10.28 (5.6)	<0.01	10.98 (6.0)
(≥36 years)	9.93 (5.74)	8.83 (5.27)	<0.01	9.62 (5.63)
Mean (SD) mature oocyte yield (All Patients)	8.58 (5.27)	7.72 (4.59)	<0.01	8.35 (5.11)
(≤35 years)	8.80 (5.35)	8.08 (4.66)	<0.01	8.62 (5.19)
(≥36 years)	8.09 (5.06)	7.08 (4.38)	<0.01	7.80 (4.9)
Number of embryos transferred (fresh)	1.91 (0.86)	1.96 (0.68)	<0.01	1.93 (0.81)
(≤35 years)	1.89 (0.84)	1.94 (0.63)	<0.01	1.91 (0.79)
(≥36 years)	1.96 (0.89)	2.00 (0.76)	<0.01	1.97 (0.85)
Mean number of oocytes/embryos frozen per cycle All patients	2.14 (3.29)	1.7 (2.8)	<0.01	2.02 (3.18)
(≤35 years)	2.32 (3.4)	1.8 (2.85)	<0.01	2.18 (3.27)
(≥36 years)	1.77 (3.03)	1.51 (2.68)	<0.01	1.70 (2.94)
Number of embryos thawed and used in FET** cycles† (All patients)	0.74 (0.97)	0.67 (0.93)	<0.01	0.74 (0.97)
(≤35 years)	0.67 (0.95)	0.57 (0.9)	<0.01	0.67 (0.94)
(≥36 years)	0.91 (1.03)	0.87 (0.99)	<0.01	0.91 (1.02)

* Statistical significance for outcomes assessed via Wilcoxon two sample test and two sample T-test

† Statistical significance assessed via the two sample T-test only

** FET - Frozen Embryo Transfer

The mean number of oocytes or embryos frozen per IVF cycle was significantly higher in rFSH-treated patients than patients treated with hMG-HP (2.14 ± 3.29 vs. 1.70 ± 2.80; p < 0.01) (Table 4).

The number of IVF cycles cancelled due to inadequate response was only reported in the Swiss dataset, and was approximately 10%.

### Pregnancy outcomes

No significant differences were observed in the mean clinical pregnancy rate as percent of cycles with oocytes retrieved (rFSH 33.04% vs. 32.04% for hMG-HP; p = 0.1037). In addition, the mean rate of pregnancies ending in spontaneous abortion as a percentage of cycles where oocytes were retrieved, were comparable between the two treatment groups (6.16% vs. 6.02% for rFSH and hMG-HP, respectively; p = 0.6625) (Table 5 and Table 6).

**Table 5:** Pregnancy outcomes - all patients

Characteristic	rFSH	hMG-HP	Chi-square	p-value
Total cycles started	22665	7965		
Number (%) of total cycles with oocytes retrieved	22,467 (99.13%)	7,958 (99.91%)	54.73	<0.0001
Cycles (%) with positive pregnancy test as percent of cycles started	7595 (33.53%)	2553 (32.05)	5.72	0.0168
Cycles (%) with positive pregnancy test as percent of cycles with oocyte retrieved	7595 (33.81% per ET)	2551 (32.06% per ET)	8.09	0.0044
Any clinical pregnancy as percent of cycles with oocyte retrieved	7423 (33.04)	2550 (32.04)	56.43	0.1037
Pregnancies ending in spontaneous abortion as percent of cycles with oocyte retrieved^†^	1303 (6.16%)	478 (6.02%)	0.19	0.6625

Note: Cycles with positive pregnancy test were defined as cycles with a biochemical pregnancy and any clinical pregnancy. Clinical pregnancies were defined based on data entries 'clinical pregnancy', 'ongoing pregnancy', 'live birth', and 'spontaneous abortion'. Pregnancies ending in spontaneous abortion were defined as those with data entries 'spontaneous abortion' and 'miscarriage'. ^† ^Due to lack of data on this outcome, only data for Germany and Denmark were considered here to calculate the percentage (N = 21,160 for rFSH subgroup; N = 7,940 for hMG-HP subgroup).

**Table 6:** Pregnancy outcomes from the oocyte-retrieved cycles - age subgroups

Characteristic	rFSH	hMG-HP	Chi-square	p-value
Total cycles started				
(≤35 years)	15422	5142		
(≥36 years)	7243	2823		
Number of total cycles with oocytes retrieved				
(≤35 years)	15,293 (99.16%)	5,137 (99.90%)	32.55	<0.0001
(≥36 years)	7174 (99.05%)	2821 (99.93%)	22.55	<0.0001
Cycles with positive pregnancy test as percent of cycles started				
(≤35 years)	5,440 (35.27%)	1,741 (33.86%)	3.40	0.0651
(≥36 years)	2157 (29.78%)	812 (28.76%)	1.01	0.3150
Cycles with positive pregnancy test as percent of cycles with oocyte retrieved				
(≤35 years)	5,438 (35.56%)	1,739 (33.85%)	4.91	0.0267
(≥36 years)	2157 (30.07%)	812 (28.78%)	1.60	0.2065
Any clinical pregnancy with positive pregnancy test				
(≤35 years)	5302 (97.50%)	1739 (100%)	44.33	<0.0001
(≥36 years)	2121 (98.33%)	811 (99.88%)	11.45	0.0007
Any clinical pregnancy as percent of cycles with oocyte retrieved				
(≤35 years)	5302 (34.67%)	1739 (33.85%)	1.14	0.2864
(≥36 years)	2121 (29.57%)	811 (28.75%)	0.65	0.4197
Pregnancies ending in spontaneous abortion as percent of cycles with oocyte retrieved^†^				
(≤35 years)	860 (6.00%)	297 (5.79%)	0.30	0.5841
(≥36 years)	443 (6.48%)	181 (6.43%)	0.0071	0.9329

Note: Cycles with positive pregnancy test were defined as cycles with a biochemical pregnancy and any clinical pregnancy. Clinical pregnancies were defined based on data entries 'clinical pregnancy', 'ongoing pregnancy', 'live birth', and 'spontaneous abortion'. Pregnancies ending in spontaneous abortion were defined as those with data entries 'spontaneous abortion' and 'miscarriage'

† Due to lack of data on this outcome, only data for Germany and Denmark were considered here to calculate the percentage (N = 21,160 for rFSH subgroup; N = 7,940 for hMG-HP subgroup).

At the time of data collection, 3,194 live births had occurred in rFSH treated patients versus 1,228 live births in the hMG-HP treated group (15.86% versus 15.51% of cycles started, respectively). The multiple pregnancy rates were also comparable; 30.8% versus 25.9% with rFSH and hMG-HP, respectively. Any comparison between the two treatment groups for multiple pregnancy rate is irrelevant as it does not take into account the number of embryos transferred. The overall multiple pregnancy rate was 29.4%.

### Safety outcomes

The absolute number of cycles ending in OHSS (severity I, II, III and hospitalisation) differed significantly between treatments, with 18.92% of rFSH cycles ending in OHSS, compared to 14.09% of hMG-HP cycles (p < 0.0001), although due to limits on the data available the OHSS data were based solely on the German dataset. There was no significant difference between treatments in the mean rate of hospitalisations due to OHSS (5.64% versus 4.75% of cycles with OHSS with rFSH and hMG-HP, respectively p = 0.2483) (Table 7). However, the mean proportion of cycles with OHSS requiring hospitalisation as a proportion of IVF cycles started was significantly higher in the rFSH group (1.07% vs. 0.67%, p = 0.002).

**Table 7:** Safety outcomes: OHSS

Characteristic	Total	rFSH	hMG-hp	Chi-square	p-value
Number of cycles started*	28063	20144	7919		
Cycles with OHSS (Severity I, II, III and hospitalisation)	4928 (17.56%)	3812 (18.92%)	1116 (14.09%)	91.64	<0.0001
-Hospitalisation due to OHSS as percent of cycles with OHSS	268 (5.44%)	215 (5.64% of cycles with OHSS)	53 (4.75% of cycles with OHSS)	1.33	0.2483
-Hospitalisation due to OHSS as percent of cycles started	268 (0.95%)	215 (1.07% of cycles started)	53 (0.67% of cycles started)	9.52	0.002

*Due to lack of data on this outcome only data from Germany were included

Logistic regression was conducted retrospectively to investigate the association between the rate of OHSS, patient age, number of oocytes retrieved and type of gonadotrophin used. Results of these analyses indicated that younger age at treatment initiation and higher number of oocytes produced are associated with a statistically increased risk of OHSS and OHSS requiring hospitalisation. Type of gonadotrophin used for oocyte stimulation was not associated with a higher risk of OHSS when the regression was adjusted for number of oocytes retrieved.

## Discussion

In today's economic climate, treatment choices are made not only on the basis of drug efficacy and safety; cost considerations, driven in part by drug dosing, also play a significant role. In this large retrospective European chart review, it has been demonstrated that in IVF (±ICSI) treatment cycles from eligible patients, cycles using rFSH (as compared with hMG-HP) yield statistically more oocytes and more mature oocytes, using significantly less IU per cycle. The difference in mean IU usage per cycle was 467.1 IU; a percentage reduction of 18.4%. Furthermore, the average number of oocytes/embryos frozen per started IVF cycle was also statistically higher in rFSH cycles compared with hMG-HP cycles. The clinical pregnancy and spontaneous abortion rates were comparable between rFSH and hMG-HP treatment groups. Of the started cycles for which the final outcome was known at the time of data collection the live birth rates were similar for both gonadotrophins.

The results obtained in the current analysis are in contrast to recent systematic reviews and meta-analyses by Coomerasamy et al [9] (which included hMG as well as hMG-HP) and Al-Inany et al [10] (which included hMG-HP only) which reported no difference in average comparative doses of rFSH and hMG-HP per IVF (±ICSI) cycle. It should be noted that the doses of rFSH used in many of the trials included in the systematic review were higher than that routinely used in clinical practice. For example, although the MERIT study [7] showed that the average dose of rFSH was lower than hMG-HP, it should be noted that the starting dose of rFSH was 225 IU/day, which is higher than most clinics outside of North America would administer [15]. The mean starting dose in the current study was 176 IU/day (± 50 IU) which is a 22% reduction on the mean starting dose for rFSH in the MERIT study [7]. The more clinically relevant median starting dose of treatment was also significantly lower in the current study for rFSH than hMG-HP (150 IU/day vs. 225 IU/day); this median dose closely reflects that described in a recent article by Trew (2007) [15].

Although conventional RCTs remain the "gold standard" for evidence-based medicine, observational data reflects populations and settings that are more relevant to clinical practice. The fact that this study used data from a 'real-world' setting, suggests that the data presented herein represent a true reflection of the relative amounts of gonadotrophin usage in clinical practice. Also, this study included a large number of patients with an excess of 30,000 IVF cycles; substantially larger than clinical studies previously conducted in this area. The results herein indicate that in everyday clinical practice, a lower IU dose of rFSH can be used per IVF (±ICSI) cycle, with clinical outcomes at least equivalent to those for hMG-HP.

One implication of these study results is that the cost of IVF treatment (±ICSI) with rFSH is likely to be significantly less than is currently estimated. Current perception is that equivalent doses of rFSH and hMG-HP are required. The current study has shown that this is not the case in real-world practice and therefore, it is possible that the higher unit cost of rFSH may be offset by the lower dosage requirement versus hMG-HP.

The results of the current study also have potential implications with regard to the recent HFEA policy on single embryo transfer (SET) to minimise the risk of multiple births from IVF [16]. Multiple births place a significant health risk on both the mother (including a higher risk of miscarriage and labour complications) and the child (including a higher risk of neonatal mortality, low birth rate, cerebral palsy and congenital abnormalities) compared to singleton births. For this reason the HFEA policy aims to reduce the UK IVF multiple birth rate to 10% by 2012 over a staged period by administering SET to the most appropriate patients. A treatment strategy that delivers a statistically higher number of mature oocytes and embryos frozen per cycle may provide a more effective treatment strategy for patients suitable for SET and therefore should be the proposed treatment of choice.

The observed absolute rates of hospitalisations due to OHSS were generally low, and comparable to previous reports [17]. However the incidence of both all OHSS and hospitalisations due to OHSS were statistically higher in cycles with rFSH than with hMG-HP. These results are in contrast to previous studies comparing rFSH with hMG-HP, including the MERIT study [7] and a systematic review by Al-Inany et al [10] which reported no significant difference in the rates of severe OHSS between treatment groups. Logistic regression analyses conducted as part of this study have demonstrated that the type of gonadotrophin was not associated with a higher risk of OHSS when adjusting for the number of oocytes retrieved. It should be noted that the current study differed from many randomised controlled trials including the MERIT study in that patients with polycystic ovary syndrome (PCO) were included in the retrospective analysis whereas PCO was an exclusion criterion for the MERIT study. PCO significantly increases the risk of OHSS [18,19], and it is hypothesised that an unequal distribution of PCO patients between the two groups in this observational data set may contribute in part to the higher rates of OHSS in rFSH treated patients. Logistic regression analyses for PCO were not possible because the cause of infertility was not reported in the German dataset.

Patient characteristics should be considered before choosing the GnRH analogue for down regulation and tailoring the gonadotrophin dose to avoid excessive stimulation. This could minimise the additional cost implications of treating OHSS.

## Limitations of the study

As with all observational studies, this study is associated with limitations including selection bias and confounding factors [20]. Other limitations particular to this study include generalisability of the data, differences in how data and outcomes were reported across different centres and countries, and local treatment regulations.

In this study, a significantly higher number of treatment cycles were conducted with rFSH compared with hMG-HP (74% of total cycles were with rFSH). However, given that there were almost 8,000 cycles in the hMG-HP group, the results remain statistically robust.

The majority of the data were obtained from a German population, which means that the overall results will be largely influenced by the German data set. Therefore, the generalisability of the data for an EU population needs to be carefully considered. There were differences in data collection practices between different centres; the reporting of pregnancy outcomes differed between the participating centres, making the analysis of pregnancy outcomes challenging and restricting the reporting of some outcomes to one or more datasets. Also, in the German dataset, IVF cycles where no oocytes were retrieved were not included which explains why the proportion of cycles with oocyte retrieval was so high (>99%). It was not possible to quantify the cancellation rate in Germany or to ascertain the reasons for treatment failure within the German data set. Furthermore, due to legal constraints in Germany and Switzerland, only three embryos can be cultured per IVF cycle. This may result in a smaller proportion of embryos being produced in Germany and Switzerland when compared to other EU countries where such constraints are not imposed, and could have implications on the clinical pregnancy rate in the fresh cycle. Furthermore in Germany and Switzerland, all embryos that survive until the embryo transfer stage must be transferred to the uterus; this may have implications on the multiple birth rate. In Denmark, elective single embryo transfer has become common practice in recent years. This is unlikely to affect the overall clinical pregnancy rate because the proportion of cycles from Denmark was small in comparison to Germany.

Eligibility criteria proposed by NICE in their 2004 clinical guideline (CG11) were used to select IVF cycles eligible for inclusion in this analysis. These criteria were employed in the absence of consensus European guidelines. Although this may be viewed as a study limitation, the IVF eligibility criteria in the four countries are comparable to those criteria suggested by NICE. The maximum age limits for IVF eligibility in each market are similar to those in the NICE guideline; these range from 40-45 years, compared with a maximum age of 39 in the NICE guideline, while in Switzerland, the median age for fertility treatment is 36 years [21]. A maximum BMI of 29 is defined in the NICE criteria, whereas there are no official criteria regarding BMI limits in the included markets, although the patient may be advised to lose weight prior to IVF if obese. Reimbursement practices differ between markets; however, three treatment cycles are reimbursed in Germany and Denmark, which is in line with NICE criteria.

The four EU countries included in this analysis had readily available datasets, unlike the UK. A separate retrospective observational chart review of UK centres is currently underway.

The study included only cycles in which the starting dose of gonadotrophin was ≤300 IU/day. This dosing restriction, which was applied equally across both rFSH and hMG-HP treatment groups, was included to capture data from normal responders, thereby excluding poor responders. It is however, possible that this has introduced some bias into the study.

It should also be noted that it was not possible to attribute the number of hospitalisations to individual grades of OHSS, due to the method of data collection in Germany. However, given the size of the current study the results presented herein are robust and reflect real-world usage of gonadotrophins in IVF.

## Conclusion

The current retrospective chart review, including patients that would be eligible for IVF under the current NICE guidelines, demonstrates that cycles with rFSH yield statistically more oocytes (and more mature oocytes), using significantly less IU per cycle, versus hMG-HP. Clinical pregnancy rates were comparable between treatment groups with no evidence of higher rates of spontaneous abortion. Of the started cycles, for which the final outcome was known at the time of data collection, the live birth rates were similar for both gonadotrophins. OHSS requiring hospitalisation was low overall but significantly higher in the rFSH group. The higher unit cost of rFSH per 75 IU compared to the equivalent IU dose of the Menopur brand of hMG-HP may be offset by the reduction of total IU dose required without a loss in efficacy outcomes. The data generated from this 'real-world' study with its unprecedented cycle size is likely to yield results that are more representative of everyday clinical practice, compared to randomised clinical studies.

## Competing interests

This study was funded by Merck Serono Limited (UK). PO and RW (United BioSource Corporation) were funded by Merck Serono Limited (UK) to analyse the data and SG and AB (Abacus International) were funded by Merck Serono Limited (UK) to prepare the manuscript. GT has received honoraria from Merck Serono for lecturing and has undertaken research funded by Merck Serono. GT did not receive any financial support for this publication.

## Authors' contributions

SB and CC were responsible for designing and coordinating the study. SB, CC, PO and RW were responsible for data collection and data analysis. All authors were responsible for data interpretation and PO and RW were responsible for statistical analysis. AB and SG were responsible for writing the manuscript and all authors were responsible for reviewing the manuscript. All authors read and approved the final manuscript.
